# A bone replacement-type calcium phosphate cement that becomes more porous in vivo by incorporating a degradable polymer

**DOI:** 10.1007/s10856-021-06555-1

**Published:** 2021-06-22

**Authors:** Akiyoshi Shimatani, Hiromitsu Toyoda, Kumi Orita, Yuta Ibara, Yoshiyuki Yokogawa, Hiroaki Nakamura

**Affiliations:** 1 Department of Orthopedic Surgery, Graduate School of Medicine, Osaka City University, 1-4-3 Asahi-Machi, Abeno-ku, Osaka, 545-8585 Japan; 2 Department of Mechanical & Physical Engineering, Graduate School of Engineering, Osaka City University, 3-3-138 Sumiyoshi-ku, Osaka, 558-8585 Japan

## Abstract

This study investigated whether mixing low viscosity alginic acid with calcium phosphate cement (CPC) causes interconnected porosity in the CPC and enhances bone replacement by improving the biological interactions. Furthermore, we hypothesized that low viscosity alginic acid would shorten the setting time of CPC and improve its strength. CPC samples were prepared with 0, 5, 10, and 20% low viscosity alginic acid. After immersion in acetate buffer, possible porosification in CPC was monitored in vitro using scanning electron microscopy (SEM), and the setting times and compressive strengths were measured. In vivo study was conducted by placing CPC in a hole created on the femur of New Zealand white rabbit. Microcomputed tomography and histological examination were performed 6 weeks after implantation. SEM images confirmed that alginic acid enhanced the porosity of CPC compared to the control, and the setting time and compressive strength also improved. When incorporating a maximum amount of alginic acid, the new bone mass was significantly higher than the control group (*P* = 0.0153). These biological responses are promising for the translation of these biomaterials and their commercialization for clinic applications.

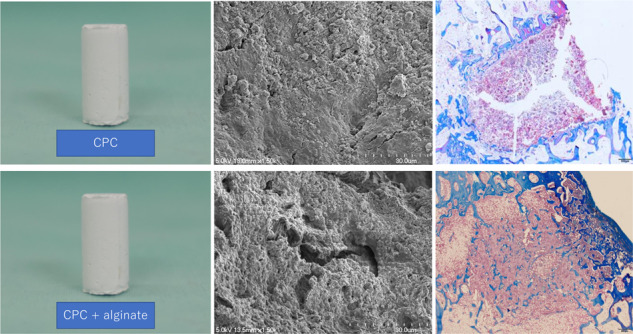

## Introduction

The use of calcium phosphate cements (CPCs) has increased in recent years, because they are useful in the direct filling of bone defects and as cell matrices in tissue engineering [1, 2]. Compared to other candidate bone replacement materials, the main merit of CPCs is their self-setting nature, which is particularly helpful when employed in the injectable form [3]. Nevertheless, CPCs suffer from several critical issues [4]. Self-setting CPCs generally have a dense microstructure. The lack of pores slows down the degradation by osteoclast-dependent resorption, which limits the potential of tissue ingrowth [5, 6]. Another main challenge is their generally poor mechanical properties [4].

To attain better mechanical and biological properties, many researchers have combined CPC with degradable polymers. Widely studied biopolymers include gelatin, collagen, chitosan, and alginate [7–11]. These biopolymers could accelerate the setting reaction while enhancing the strength of the CPC network. The setting time is especially important in clinical use [12]. The cure time of CPC is also pH dependent, and premature solidification has been reported under acidic conditions [13].

This study evaluated a series of CPC-alginate compounds. Alginate is a naturally derived polysaccharide that has been used in many biomedical applications, including cell encapsulation, drug delivery, and wound dressing because of its nontoxicity, degradability, and cross-linking ability [11, 14]. However, compared to other biopolymers, there have been only limited studies of alginate in conjunction with CPCs [10, 15], particularly for systematic investigation of the mechanical properties and in vivo performance. Furthermore, we are not aware of any previous experiments that considered enhancing CPC with low viscosity alginic acid, which has a much higher solubility than the medium viscosity one.

The present study aims to incorporate low viscosity alginic acid into CPC as a self-setting bioactive cement. The composition, morphology/microstructure, setting time, compressive strength, surface reaction, and degradation of CPC-alginate composites and the associated bone healing were investigated in vitro and in vivo. Our main hypothesis is that the introduction of low viscosity alginic acid causes the formation of a series of interconnected macropores that facilitate the entry of bone marrow cells and new bone formation.

## Materials and methods

### In vitro study and characterization

#### Preparation of CPC-containing polymer complex

Tetracalcium phosphate (TTCP) powder was synthesized as follows. A calcium hydroxide solution (Nacalai Tesque Inc., Japan) was added dropwise into a phosphoric acid solution (Wako Pure Chemical Industries, Ltd., Japan) that was kept below 10 °C, and the solution was stirred at room temperature for 24 h. The precipitate was filtered, dried, and heated at 1500 °C for 5 h, ground in an agate mortar, and then sieved under 75 μm to obtain the TTCP powder (average particle size: 21.5 µm). Commercial dicalcium phosphate anhydrous (DCPA) powder (Taihei Chemical Industrial Co., Ltd., Japan) was ground in a ball mill with pure water and zirconia balls for 48 h to obtain the DCPA powder (average particle size: 0.52 µm). Equal molars of TTCP powder and DCPA powder were mixed in a blender and with an agate mortar to form the CPC powder.

A mixed solution of dipotassium hydrogen phosphate and potassium dihydrogen phosphate (0.2 and 0.1 g, respectively, both from Kishida Chemical Co., Ltd., Japan) was prepared with 10 mL of Milli-Q water. Low viscosity sodium alginate (UIV-L3, viscosity 20–50 mPa · s, KIMICA Co., Japan) was used as a biocompatible polysaccharide and gelation agent to develop the nonrigid CPC. Four liquids (Liquid 0 to Liquid 3) were prepared with 0, 5, 10, and 20 wt% low viscosity sodium alginate, respectively. The CPC powder was mixed with each curing liquid at a powder/liquid ratio of 3, and the mixture was placed in a cylinder (Table 1). The corresponding hardened specimens were denoted CPC0–CPC3.

**Table 1:** Composition of CPC

	Liquid 0 (CPC0)	Liquid 1 (CPC1)	Liquid 2 (CPC2)	Liquid 3 (CPC3)
Milli-Q water (g)	10	10	10	10
Alginic acid (g)	0	0.5	1	2
K2HPO4 (g)	0.2	0.2	0.2	0.2
KH2PO4 (g)	0.1	0.1	0.1	0.1

#### Characterization

The pH of the prepared curing liquid was measured using a pH meter (D-51, HORIBA, Ltd., Japan). The pH meter was first calibrated using three points measured in phosphate pH standard solution (pH 9.18), neutral phosphate pH standard solution (pH 6.86), and phthalate pH standard solution (pH 4.01), all from Wako Pure Chemical Industries, Ltd., Japan. Five replicates were measured for each liquid, and the average value is reported.

The setting time was measured according to JIS (Japanese Industrial Standards) T0330-4. Kneaded CPC paste was placed in a cylindrical Teflon mold (inner diameter: 6 mm, height: 12 mm) within 90 s after the start of kneading. The mold was placed in a container that also held a sponge soaked in Milli-Q water in an incubator held at 37 °C. Setting of the material was monitored using a Vicat needle (A-004, MECC Co., Ltd, Japan) with a 1 mm^2^ tip at 300 g load. Every 30 s, the Vicat needle was placed on the surface of the specimen, and the specimen was considered set when the needle failed to make a perceptible circle on its surface. The test was performed in triplicates for each condition, and the average value was reported as the setting time.

The compressive strength was determined in accordance with JIS T0330-4. The mixture of CPC powder and liquid was placed in the hole of a Teflon mold (diameter: 6 mm, height: 12 mm). The mold was stored in an incubator at 37 °C and 100% humidity for 1 h, and then immersed in Milli-Q water for 1 day. The diameter of each specimen was measured using a micrometer (M300, Mitsutoyo, Japan). Then, the specimen was individually placed on the stage of a universal tester (Autograph AGI-20kN, Shimadzu Co., Japan). Measurement was carried out at a head speed of 0.500 mm s^–1^ and a sampling interval of 100 ms. Five specimens from each type of CPC were used, and each specimen was measured ten times. Data analysis was performed using control/analysis software (Trapezium, Shimadzu Co., Japan), and the average value was used as the compressive strength.

Scanning electron microscopy (SEM) analysis employed an S-4700 (Hitachi Ltd., Japan) instrument at an accelerating voltage of 5.0 kV under a pressure of 1.00 × 10^−3^ Pa or less, on samples coated with 12 nm of Pt–Pd using an ion sputter (E-1030, Hitachi Ltd., Japan). The mixture of CPC powder and liquid was placed in a cylindrical Teflon mold (diameter: 6 mm, height: 12 mm). Each mold was stored in an incubator at 37 °C and 100% humidity for 1 h, and then immersed in acetic acid–sodium acetate buffer (see details below) for 7 days. The diameter of each specimen was measured using a micrometer (M300, Mitsutoyo, Japan). For the buffer, first two solutions were prepared using Milli-Q water: one containing 0.08 mol L^–1^ acetic acid (Kishida Chemical Co., Ltd. Japan) and the other containing 0.08 mol L^–1^ sodium acetate (Kishida Chemical Co., Ltd., Japan). Then, the two solutions were mixed at a ratio of 1:7 to prepare the buffer, which had a pH of 5.50 ± 0.02.

### In vivo studies

#### Surgery

**Fig. 1:**
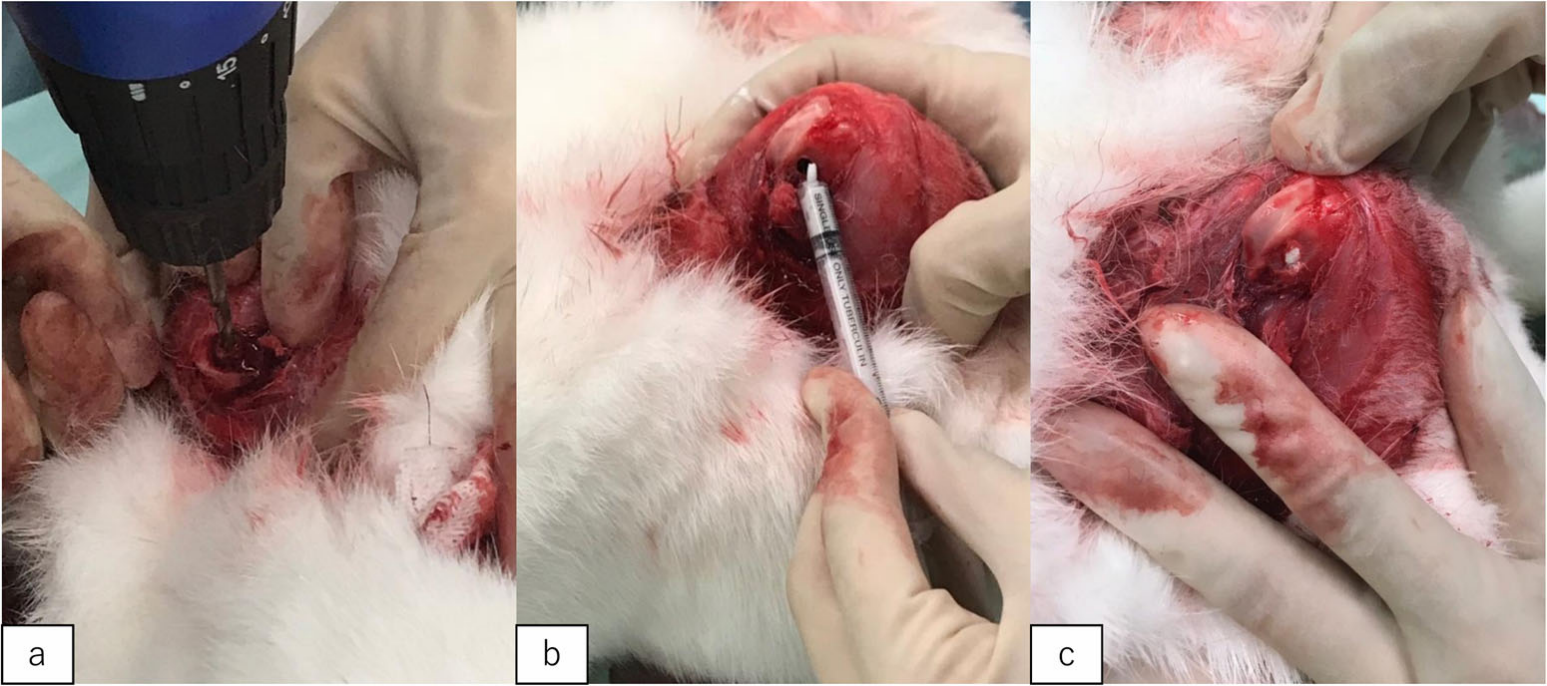
Photographs of the operation. **a** Preparation of a bone hole (diameter: 4 mm, depth: 10 mm) outside the condyle of the rabbit femur. **b**, **c** Material was injected into the bone hole using a syringe

The animal protocol was approved by the Animal Ethics Committee of Osaka City University. Ten retired female New Zealand white rabbits weighting 4–4.5 kg were used for implantation of CPC0 (control), CPC1, CPC2, and CPC3. Five femurs were used in each group. The rabbits were anesthetized with ketamine hydrochloride, and a cylindrical defect (diameter: 4 mm, depth: 10 mm) was drilled on both femur condyles using a trephine drill. A CPC sample was injected into the defect site, which was then sutured (Fig. 1). After 6 weeks, the rabbits were sacrificed, and the femur condyles were obtained for analysis. The surgery was performed by two authors (AS and KO).

#### X-ray and micro-CT analysis

X-ray images were taken for each femur condyle immediately after surgery as well as 3 and 6 weeks later. The extracted rabbit femur condyles were fixed in 10% neutral buffered formation solution at room temperature. Micro-CT was done using inspeXio SMX-90CT Plus (Shimadzu Corporation, Japan). The scan data were reconstructed using 3D image processing software (ExFact VR, Nihon Visual Science, Inc., Japan).

#### Histological analysis

After the micro-CT analysis, each sample was decalcified with Morse solution (Wako Pure Chemical Industries, Ltd., Japan) and dehydrated using an alcohol series. Residual alcohol was removed by immersion in xylene. After embedding the tissue sample in paraffin block, 4-µm-thick slices were cut using a microtome and treated with hematoxylin and eosin (H&E) and Masson’s trichrome stains. The sections were observed using a model BX53F microscope (Olympus, Japan) and photographed with an Olympus DP74 camera. The images were analyzed using Cellsens software (Olympus, Japan). The area of new bone mass was measured using ImageJ based on Masson’s trichrome staining. The total area of new bone was measured in a 100-fold visual field at three locations: on the cortical bone side, the central side, and the cancellous bone side of the bone defects. Image analysis and measurement of the total area of new bone were conducted by two authors (HT and HN) in a blinded situation.

### Statistical analysis

Statistical analysis was performed using the Excel Statistics software for Windows (version 2019; SSRI Co. Ltd., Tokyo, Japan). Data were expressed as mean ± standard deviation. The data analysis used one-way analysis of variance followed by a multiple comparison using the Tukey test. *P* values < 0.05 were considered statistically significant.

## Results

### In vitro properties

Table 2 shows the pH, setting time, and compressive strength for different curing liquids or CPCs. The pH of the curing liquids was significantly reduced after adding sodium alginate. Liquid 3 with 20 wt% alginic acid showed the lowest pH (7.00 ± 0.05), which is statistically significantly lower than that of Liquid 0 (7.21 ± 0.05, *P* < 0.001). At the same time, the setting time of CPC was shortened with increasing content of alginic acid, from 56 ± 4.6 min for CPC0 to 11.5 ± 0.5 min for CPC3. Therefore, the curing liquid with the lowest pH (Liquid 3) resulted in the shortest setting time, with statistically significant difference compared to the control (*P* < 0.001). The compressive strength also significantly increased after adding sodium alginate compared to the control. CPC3 showed the highest compressive strength of 46.7 ± 8.6 MPa, whereas CPC0 showed the lowest (7.3 ± 2.1 MPa). Statistically, CPC3 was significantly stronger than CPC0 (*P* < 0.001).

**Table 2:** pH, setting time and compressive strength

	Liquid 0 (CPC0)	Liquid 1 (CPC1)	Liquid 2 (CPC2)	Liquid 3 (CPC3)
pH	7.21 ± 0.05	7.05 ± 0.04*	7.11 ± 0.03*	7.00 ± 0.05*
Setting time (min)	56 ± 4.6	20 ± 1.7*	17 ± 2.6*	11.5 ± 0.5*
Compressive strength (MPa)	7.3 ± 2.1	16.3 ± 3.7	34.9 ± 4.1*	46..7 ± 8.6*

*P  < 0.05, each liquid/CPC group versus Liquid 0/CPC0 group

**Fig. 2:**
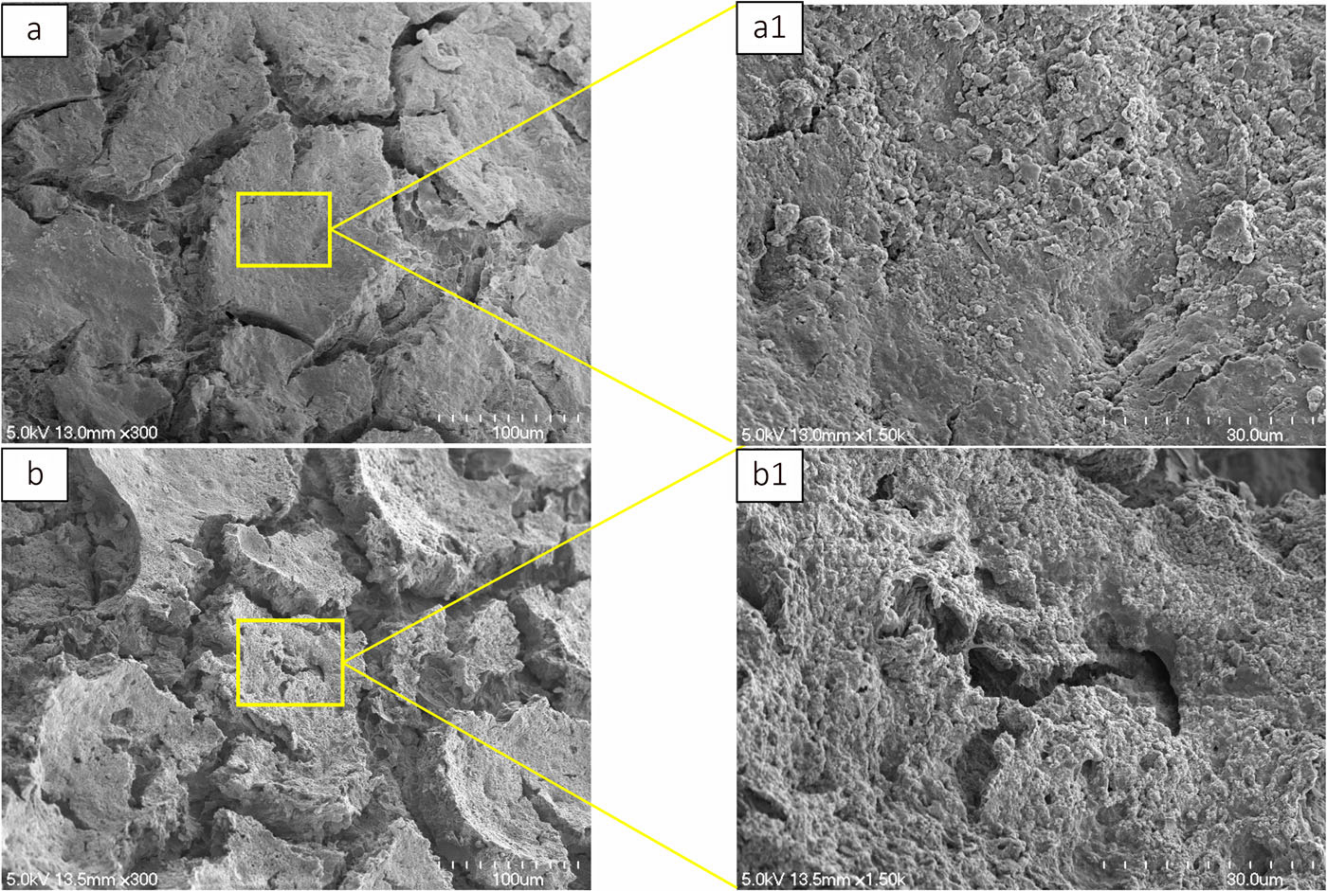
SEM micrographs. Cross-sectional microstructures in the set CPC samples **a** without and **b** with alginate (20 wt%). Little porosity was detected in CPC sample without alginate **(a1**). Adding alginate resulted in the formation of macropores within the bulk materials (**b1**)

Figure 2a is a representative low-magnification SEM image of CPC0, and Fig. 2a1 is the high-magnification counterpart. The corresponding images for CPC3 are displayed in Fig. 2b and Fig. 2b1, respectively. In the low-magnification image, the alginic acid-containing CPC3 shows more pores and a lower density than the control (CPC0). The high-magnification images further indicate that the CPC3 polymer matrix has more large pores than CPC0.

### In vivo results

**Fig. 3:**
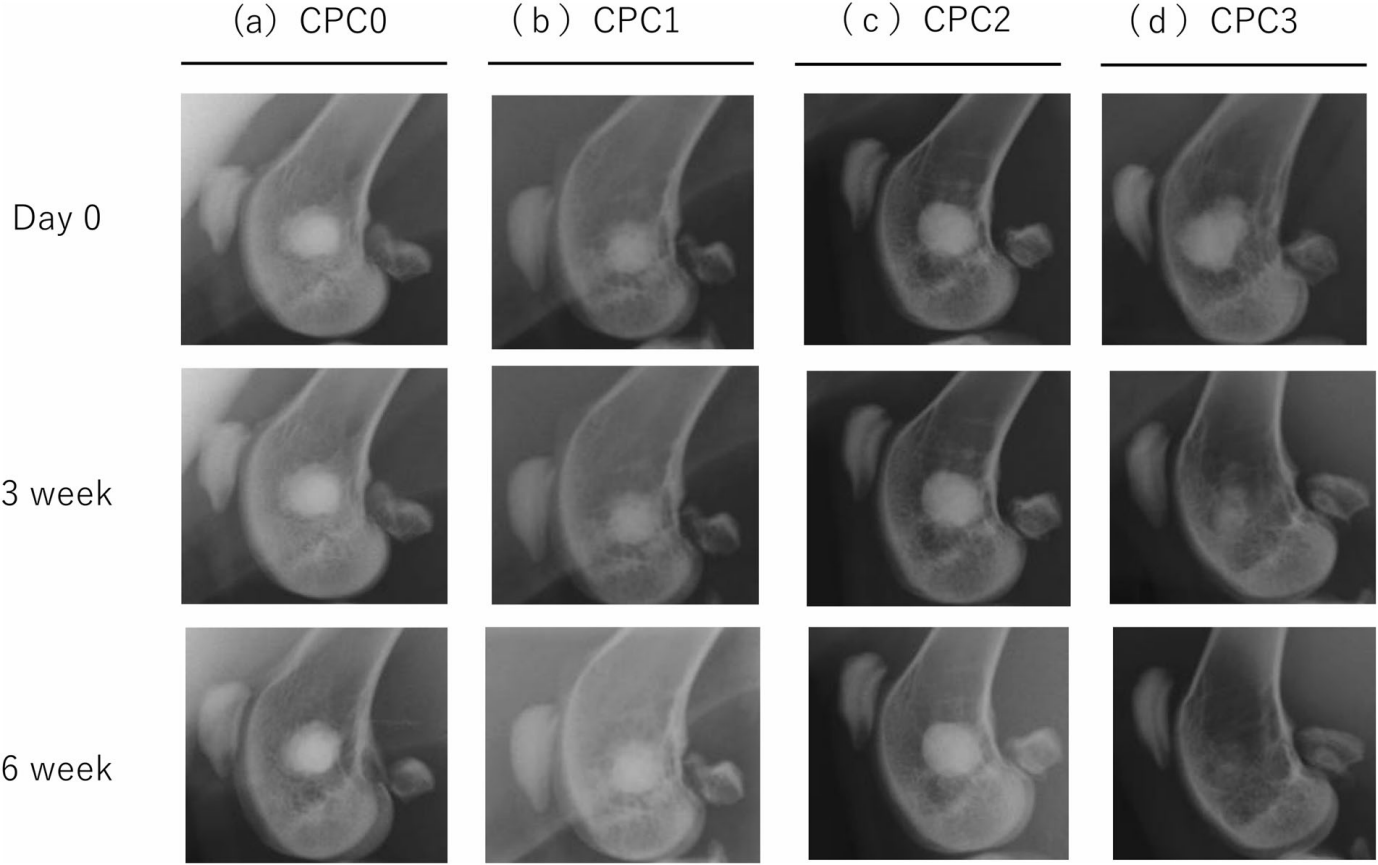
X-ray images of CPCs at 0 day, 3 weeks, and 6 weeks after surgery. Impaired portion of femoral condyle indicates the CPCs. Only CPC3 displays a time-dependent increase in permeability at the site. This is especially noticeable in the image after 6 weeks

Representative X-ray images are presented in Fig. 3. Defect bridging was monitored by serial radiography at 0, 3, and 6 weeks after surgery. At 3 weeks, we observed more elaborate structures and extensive absorption at the defect site treated with CPC3 compared with the other groups. At 6 weeks, bone regeneration of marrow space was visible in the group treated with CPC3.

**Fig. 4:**
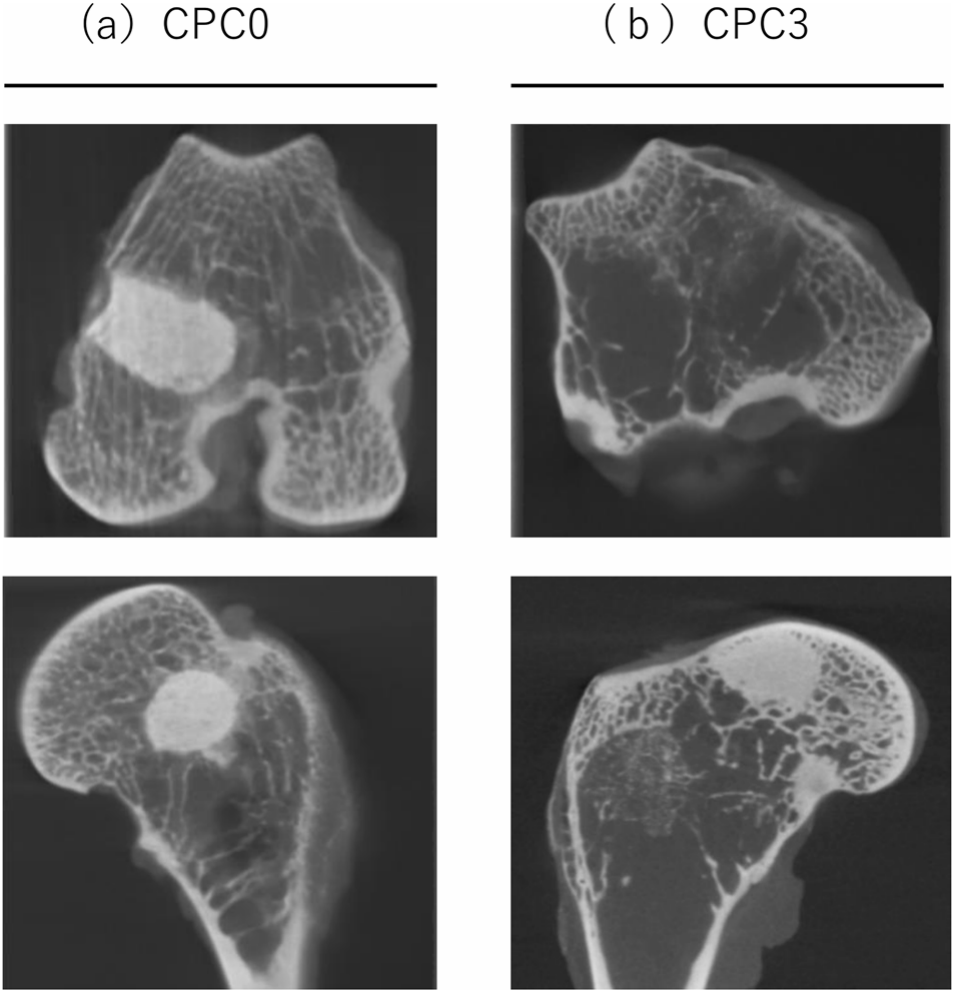
Micro-CT images of CPC0 and CPC3. Upper part: axial image, lower part: sagittal image. CPC3 shows lower brightness in the bone tunnel than CPC0

Six weeks after the operation, the rabbits were sacrificed, and all treated femurs were imaged by micro-CT. Similar to the X-ray images, no CPC degradation was observed in the CPC0 group, whereas the CPC3 group showed degradation and bone formation at the defect periphery. This result suggests that early bone replacement occurred, and there may be bone ingrowth into the CPC3 implant at 6 weeks (Fig. 4).

**Fig. 5:**
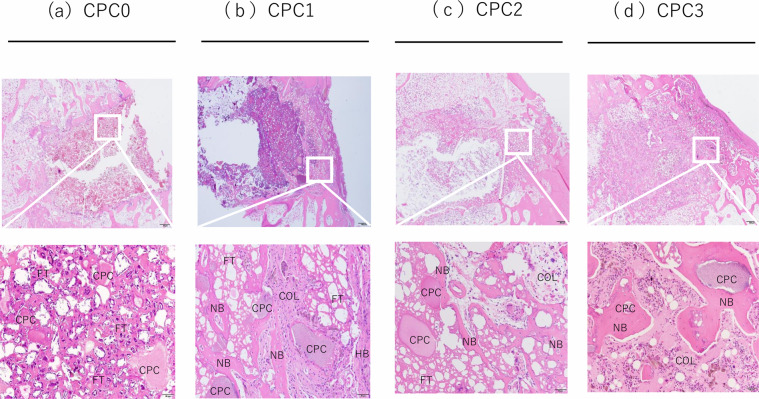
Histological micrographs of tissue sections stained with hematoxylin and eosin. Implant interface of CPC0–CPC3 after 6 weeks of implantation in rabbit femoral condyle. The images show axial cross section through the center of the bone hole. NB newly formed bone, HB host bone, COL collagen, FT fibrous tissue

**Fig. 6:**
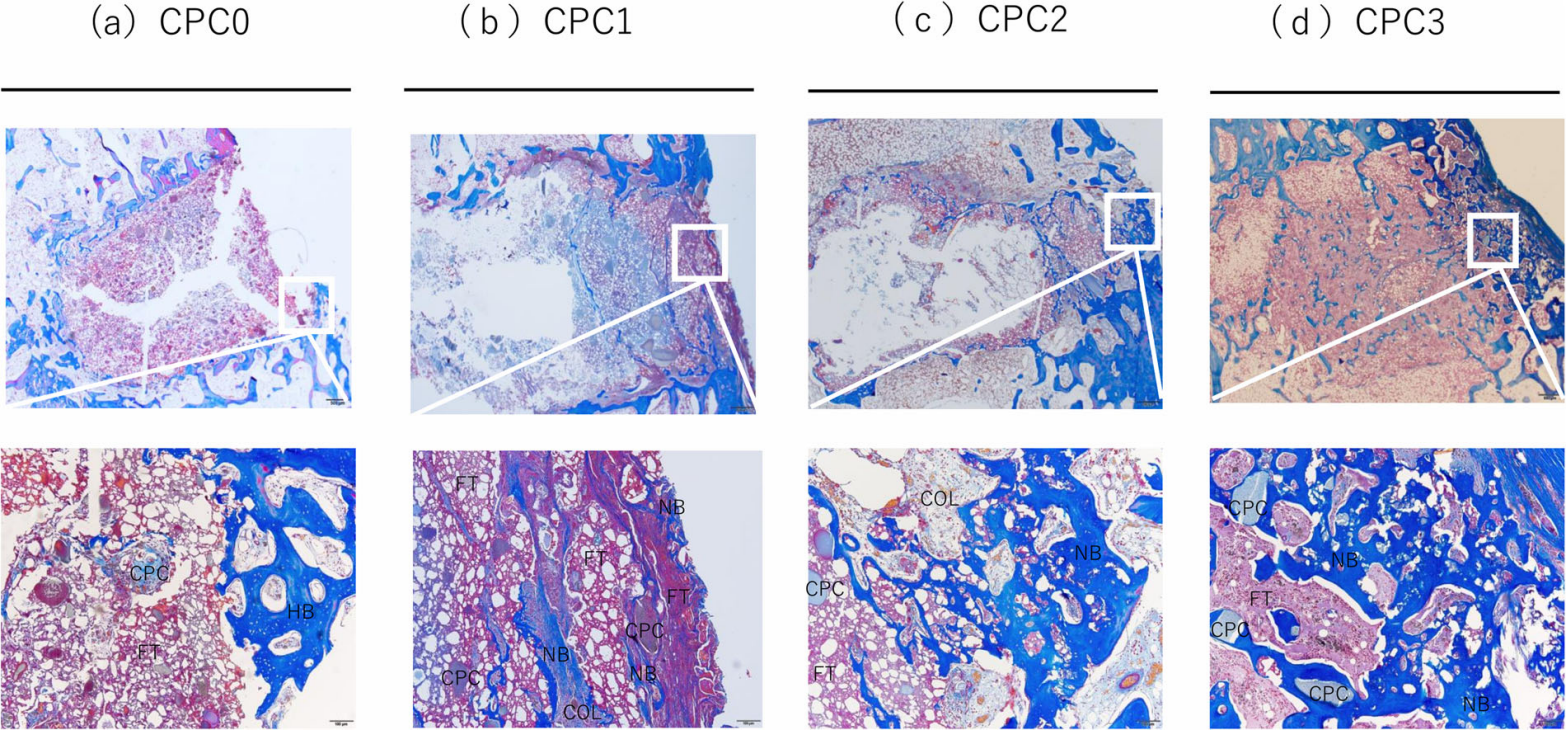
Histological micrographs of tissue sections stained with Masson’s trichrome. Masson’s trichrome-stained sections of CPC0–CPC3 after 6 weeks of implantation in rabbit femoral condyle. Images show axial cross section through the center of the bone hole. NB newly formed bone, HB host bone, COL collagen, FT fibrous tissue

Figures 5 and 6 display representative images of sections stained with H&E and Masson’s trichrome stain, respectively. After 6 weeks, the accumulation of nucleated cells around the CPC0 implant was poor. In contrast, the implants containing alginate (CPC1–CPC3) showed accumulation of nucleated cells and new bone formation around them. Most of the gaps in CPC0 were covered with fibrous tissue, however in CPC1–CPC3 there was much collagen tissue (Fig. 5). Complete cortical bridging was observed in CPC1–CPC3, while no apparent cortical bridging was observed in CPC0. CPC3 further showed apparent new bone formation within the implant (Fig. 6).

**Fig. 7:**
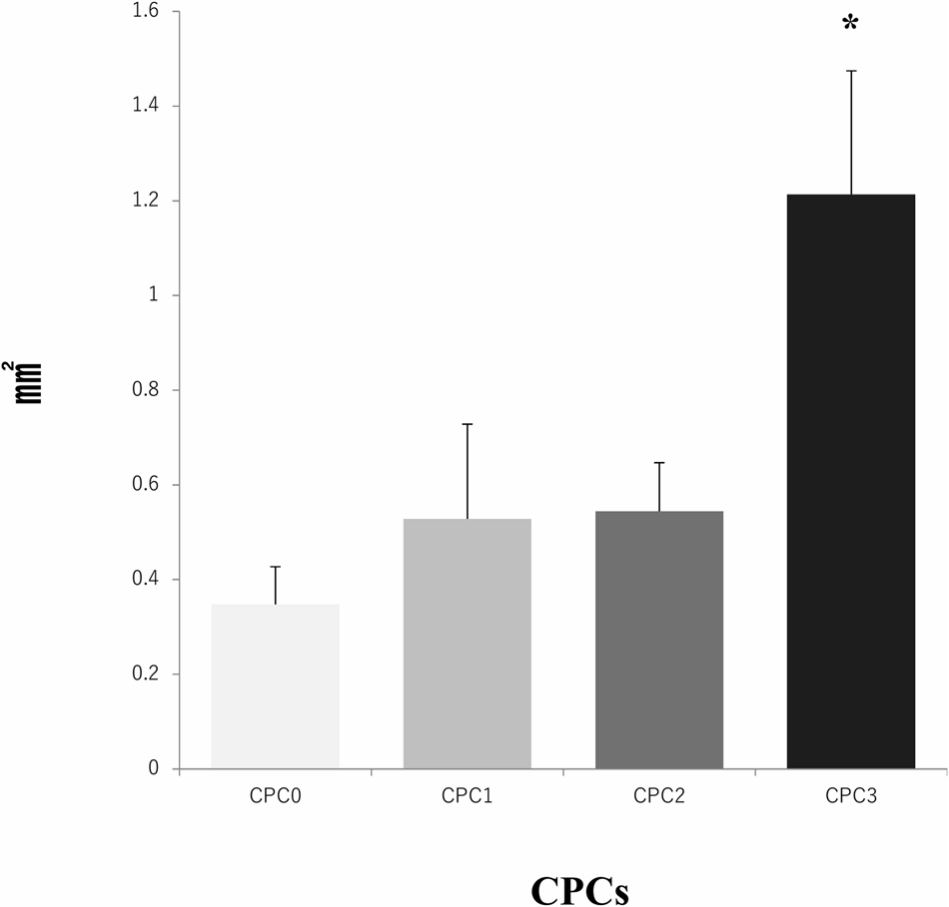
Area of new bone in the bone tunnel. The area was measured using ImageJ and based on the tissue after Masson’s trichrome staining. A higher alginic acid content in the CPC resulted in a larger new bone area. *CPC3 had a statistically significant increase in compression strength compared to CPC0 (*P* = 0.0153)

According to the area of new bone in the bone hole measured after Masson’s trichrome staining, CPC3 resulted in a larger amount of new bone than the CPC0 group with statistical significance (*P* = 0.0153, Fig. 7).

## Discussion

This study demonstrated that the incorporation of low viscosity alginic acid caused a porous CPC (Fig. 2). Moreover, the setting time was reduced and the compressive strength increased (Table 2). When this composite was implanted in an animal model, there was extensive new bone formation with gradual degradation of CPC (Figs. 4–7). This is the first study to show that low viscosity alginic acid enhances the biological performance of CPCs.

Injectable CPC is widely used for repairing bone defect and bone augmentation due to the minimal invasion required [16, 17]. A number of polymers (chitosan, gelatin, hyaluronic acid, methylcellulose, and others) have been added as viscous binders to improve the biological performance of CPC materials and their injectability [18]. In this study, we investigated the effect of adding different amounts of low viscosity sodium alginate on the CPC’s setting time, mechanical properties in vitro, and the biocompatibility and bone replacement rate in vivo (rabbit femur). From the results, the injectable CPC3 composite containing 20 wt% alginate showed degradation and subsequent bone replacement, with significant advantages over pure CPC and other composites with less alginate.

Specifically, we used a low viscosity sodium alginate (viscosity 20–50 mPa · s, molecular weight about 40,000–50,000). As far as we know, low viscosity alginic acid has not been applied in the curing liquid for CPC before. When its amount exceeds 20 wt%, the liquid will gel strongly, and kneading it with CPC would be difficult. Thus, the limit was set at 20 wt%. For the more commonly used medium viscosity alginic acid (sodium alginate 80–120, viscosity 80–120 mPa · s, molecular weight about 64,000, Wako Pure Chemical Industries, Ltd.), the maximum dissolution amount is only 2 wt% (data not shown). Therefore, a much larger amount of polymer (6.4–7.8 times by weight) could be dissolved when using the low viscosity sodium alginate.

Adding sodium alginate to CPC produced several benefits. First, it reduced the average setting time from 56 min in CPC0 to 11.5 min in CPC3, representing a reduction of 80%. In actual surgeries, a shorter curing time is advantageous [19]. It has been reported that under acidic conditions, the solubility of CPC increases and premature solidification of CPC is likely [13]. In this study, we also observed a lower pH when increasing the content of alginic acid (possibly due to its chelating effect), which is thought to shorten the setting time. The setting of CPC proceeds via dissolution–precipitation reactions, similar to the setting reactions of gypsum [20–22]. In the case of α-tricalcium phosphate (α-TCP, α-Ca_3_(PO_4_)_2_)-based cement, α-TCP dissolves to supply Ca^2+^ and HPO_4_^2−^. This liquid phase would be supersaturated with respect to calcium deficient hydroxyapatite (cdHAp; Ca_9_(HPO_4_)(PO_4_)_5_(OH)), leading to the precipitation of cdHAp. The precipitated cdHAp crystals then interlock with each other to form a set mass. Acceleration of either step mentioned above could theoretically accelerate cdHAp formation, thereby shortening the setting time of CPC [23].

Second, CPC3 with a maximum amount of sodium alginate had a compression strength 6.4 times that of the control (average values: 46.7 versus 7.3 MPa). It was reported that cdHAp on the surface of α-TCP affects not only the setting time but also the mechanical strength of the set CPC. When cdHAp is present on the surface of α-TCP powder, the Ca^2+^ and HPO_4_^2-^ ions supplied by α-TCP would be used for the growth of cdHAp crystals and facilitate their interlocking [23]. The increased strength is attributed to the addition of high molecular weight polymer, as well as ionic cross-linking (a characteristic of alginic acid). This increased strength with added alginic acid is certainly superior to that of cancellous bone (2–12 MPa), but not that of cortical bone (100–230 MPa). Hence, this CPC compound is more suitable for nonloaded surfaces.

Third, 6 weeks after implanting CPC3 containing 20 wt% alginic acid, early bone replacement was observed on the X-ray and micro-CT images. The area of new bone was 3.5 times that of the CPC0 group. Although CPC is a convenient injectable bone replacement material, it is difficult to formulate into specific shapes that are useful for tissue engineering [24]. However, Lee et al. reported that a moist environment filled with alginic acid in the CPC space tends to form 3D scaffolds with complex shapes, providing a 3D matrix suitable for tissue cells to adhere and spread [25]. In this study, we also immersed CPC in an acetate–sodium acetate buffer prior to SEM imaging, in order to observe the surface changes in a similar environment. Activated osteoclasts express many proton pumps in the cell membrane on their bone surface side, producing an acidic region with pH = ca. 4.7–6.8 on the bone surface [26]. The SEM images confirmed that the alginic acid-containing CPC was more porous than the control. It is thought that the bone replacement action is enhanced when the porosity increases. Also, the porous microstructure allows bone marrow cells to migrate into the CPC composite. Extensive new bone formation with gradual degradation of CPC was observed in this composite. In terms of mechanical strength, we speculate that the slow biodegradation and excellent osteoinductivity are important factors for the synthetic biomaterials to repair bone defects. Such injectable CPC materials containing low viscosity alginic acid may have great potentials in bone regeneration, with significant clinical advantage over the simple CPC. This is an attractive option in the field of bone tissue engineering, especially for the minimally invasive technique. A future technology for mixing any type of alginic acid with the curing solution may allow more precise control over the size of interconnected pores in CPC and its degradation rate. Another interesting possibility is using some kind of degradable polymer loaded with multiple drugs, growth factors, etc. to enhance angiogenesis and osteogenesis in challenging environments such as concomitant infection, poor blood supply, and huge bone defects.

## Conclusions

Low viscosity alginic acid not only possesses good biocompatibility and degradability, but also causes faster and more effective osteogenesis when combined with CPC for bone repair. In this study, combinations of CPC and alginic acid demonstrated a reasonable setting time, suitable mechanical strength, and excellent degradability and bioactivity. Since it is prepared in the paste form, this composite can be easily injected. Such cement could become good artificial bone material for clinical applications.

## Data Availability

Data supporting the findings of this study are available within the article.
